# Alveolar Tissue Fiber and Surfactant Effects on Lung Mechanics—Model Development and Validation on ARDS and IPF Patients

**DOI:** 10.1109/OJEMB.2021.3053841

**Published:** 2021-01-22

**Authors:** Jiayao Yuan, Caitlyn M. Chiofolo, Benjamin J. Czerwin, Nikolaos Karamolegkos, Nicolas W. Chbat

**Affiliations:** Columbia University 5798 New York NY 10027 USA; Quadrus Medical Technologies New York NY 10001 USA; Quadrus Medical Technologies New York NY 10001 USA; Columbia University 5798 New York NY 10027 USA

**Keywords:** Alveolar compliance, ARDS, IPF, pulmonary fibers, pulmonary surfactant concentration

## Abstract

*Goal:* Alveolar compliance is a main determinant of lung airflow. The compliance of the alveoli is a function of their tissue fiber elasticity, fiber volume, and surface tension. The compliance varies during respiration because of the nonlinear nature of fiber elasticity and the time-varying surface tension coating the alveoli. Respiratory conditions, like acute respiratory distress syndrome (ARDS) and idiopathic pulmonary fibrosis (IPF) affect fiber elasticity, fiber volume and surface tension. In this paper, we study the alveolar tissue fibers and surface tension effects on lung mechanics. *Methods:* To better understand the lungs, we developed a physiology-based mathematical model to 1) describe the effect of tissue fiber elasticity, fiber volume and surface tension on alveolar compliance, and 2) the effect of time-varying alveolar compliance on lung mechanics for healthy, ARDS and IPF conditions. *Results:* We first present the model sensitivity analysis to show the effects of model parameters on the lung mechanics variables. Then, we perform model simulation and validate on healthy non-ventilated subjects and ventilated ARDS or IPF patients. Finally, we assess the robustness and stability of this dynamic system. *Conclusions:* We developed a mathematical model of the lung mechanics comprising alveolar tissue and surfactant properties that generates reasonable lung pressures and volumes compared to healthy, ARDS, and IPF patient data.

## I. Introduction

The main function of the lungs is to provide freshly breathed oxygen (O_2_) to the blood capillaries, while taking carbon dioxide (CO_2_) in exchange from them and expelling it to the atmosphere. It does so tidally via repetitive inspiratory and expiratory cycles. This exchange is possible thanks to a hydraulic and a diffusive transport mechanism. Hydraulically, the respiratory system is defined as one tracheobronchial tree, that has 24 generations of dichotomous branching, extending from the trachea (close to the mouth) down to the alveolar sacs [1]. Generation 0 (trachea) to generation 16 (terminal bronchioles) are conducting pipes, known as dead space, where no gas exchange takes place. The branches from the respiratory bronchioles to the alveolar sacs (generation 17 to 23) are defined as transitional and respiratory zones where CO_2_ in the blood is exchanged for O_2_ in the air diffusively.

Lung parenchyma, comprising a large number of thin-walled alveoli, has a complex internal structure with an inner surface area that maximizes gas exchange. The alveolus the basic gas exchange unit, is lined with a layer of epithelial cells (type I and type II). Type II epithelial cells secrete surfactants that are a mixture of lipids and proteins that line the inside of the alveoli, forming a film that reduces surface tension, to keep alveoli open, hence preventing alveolar collapse (atelectasis) and facilitating respiration. Alveolar surface tension is generated from molecular attractive forces of water on alveoli tissue. The surfactant plays a critical role in maintaining lung elasticity by lowering those attractive forces, effectively reducing surface tension [2], [3]. Low surfactant concentration keeps alveoli closed at low lung pressure range (lung threshold opening pressure increases) due to the alveoli's inability to withstand increased surface tension. In between epithelial cells and the capillary basement membrane is the extracellular matrix (ECM) of the alveolar septal wall. The ECM contains elastin and collagen that determine the elasticity of the pulmonary tissue. Elastin is an essential load-bearing component of the ECM, and can withstand a large range of strain. Collagen, a helical shaped protein, provides considerable recoil stress during stretching. When lung volume increases to a certain level, the stress of the lung tissue increases significantly due to the nonlinear stress-strain relation of the collagen. Considering all these effects, the alveoli are held open under the balance of three pressures: 1) the transmural pressure, which is the difference between pleural cavity pressure and alveolar pressure, 2) the stresses in the elastin and collagen fibers, and 3) the alveolar surface tension, as determined by the surfactant concentration. The balance of these three pressures plays a crucial role in patients with respiratory distress. Patients with acute respiratory distress syndrome (ARDS), idiopathic pulmonary fibrosis (IPF) have severely impaired gas exchange [4]–[9], due to increased lung stiffness that could cause alveolar collapse. Poor gas exchange causes hypoxemia, low levels of oxygen in the blood, that would lead to tissue and organ failure. Studies have shown that patients with ARDS, caused by pneumonia, sepsis, chest injury, etc., have low surfactant concentration and an increased amount of collagen compared to a healthy population [4]–[6]. On the other hand, patients with IPF were identified as not only having an increased amount of collagen but also a degraded quality of collagen [7]–[9]. ARDS and IPF patients have deficient pulmonary compliance and experience shortness of breath. Severe cases are life-threatening and need exogenous breathing support, like a mechanical ventilator. Pressure vs volume (PV) curves have been used at times at the patient's bedside [10] to show the stiffness of the diseased lungs, and it is crucial to recruit the collapsed alveoli in order to improve gas exchange.

Understanding the pulmonary system is studying lung mechanics, alveolar elasticity, gas exchange, as well as respiratory muscles and ribcage mechanics. Our focus here, however, is on lung mechanics and alveolar elasticity. The proposed lung model calculates alveolar compliance in time as a function of surfactant concentration, lung fiber (elastin and collagen) quantity, and fiber quality. Lung mechanics variables (*e.g.,* alveolar pressure) can then be computed using this time-varying alveolar compliance, and lung resistances. We validate the model via ARDS and IPF patients’ data and PV curves. Furthermore, a study by, Gattinoni [11] claims that 20-30% of the coronavirus disease (COVID-19) patients admitted to the intensive care unit have severe hypoxemia associated with low lung compliance values. The proposed model thus has the potential to simulate COVID-19 patients who are lung compliance compromised.

In what follows, we first provide a brief literature review of the mathematical models of the respiratory system. We then describe the development of the proposed lung model (modeling approach, equations, and parameters). We present simulation results and compare them to healthy human data [12], a published lung mechanics model [13], ARDS patient data and IPF patient data [17]. Finally, we summarize the model performance, and highlight future extensions of this work.

## II. Model Development

Lung mechanics models with varying levels of rigor have been developed by researchers. A linear one-compartment (balloon type) dynamic model of the respiratory system with one resistive element ($R$) and one capacitor ($C$) is well accepted by the clinical community due to its simplicity [18]. A few mechanical ventilator applications adopt such a model to assess the patient's pulmonary health status by estimating $R$ and $C$ [19], [20]. In 1991, a more rigorous linear model was proposed by Rideout [21] that included four compartments: larynx, trachea, bronchi, and alveoli. In his work, lung air tubes that share similar geometric and functional properties were lumped into one compartment. Rideout's model adequately describes lung mechanics, but fails to include (the nonlinear) alveolar elasticity and dynamic compliance, both of which are included in our work. Further, a complex model with several (10-50) parallel lung sections can also be found in the literature [22]. This work describes each section with an analog electrical network of a resistance in series with a capacitor.

To describe the nonlinear behavior of alveolar compliance, Venegas *et al*. proposed a sigmoidal equation to represent lung pressure – volume relationship [23]. This equation fits well to inflation and deflation limbs of the PV curves of normal and diseased lungs. Denny and Schroter developed a series of finite element models for the mammalian lung alveolar duct [24]–[27]. In their models, alveolus geometry was considered as a truncated octahedron and the amount and distribution of elastin and collagen fiber bundles were studied. Surface tension effects as a function of surfactant concentration were fitted from available published patient data [28], [29]. Finally, Fujioka *et al*. developed a lung parenchyma model [30], which comprises individual alveoli. In that work, alveolar deformation was computed based on the elastin and collagen stresses, surface tension, and transmural pressure. Fujioka *et al*. focused on the effect of surfactant on the tethering force that is applied on the alveoli. To validate the model, Fujioka *et al*. simulated ARDS and compared their simulation results to the sigmoidal functions proposed by Venegas *et al*. [23]. We build upon Fujioka's and Venegas's by modeling the lung mechanics from the elasticity of individual alveolar units and their contribution to the time-varying alveoli capacitance, while including additional lung mechanics compartments per Rideout.

**Fig. 1.**
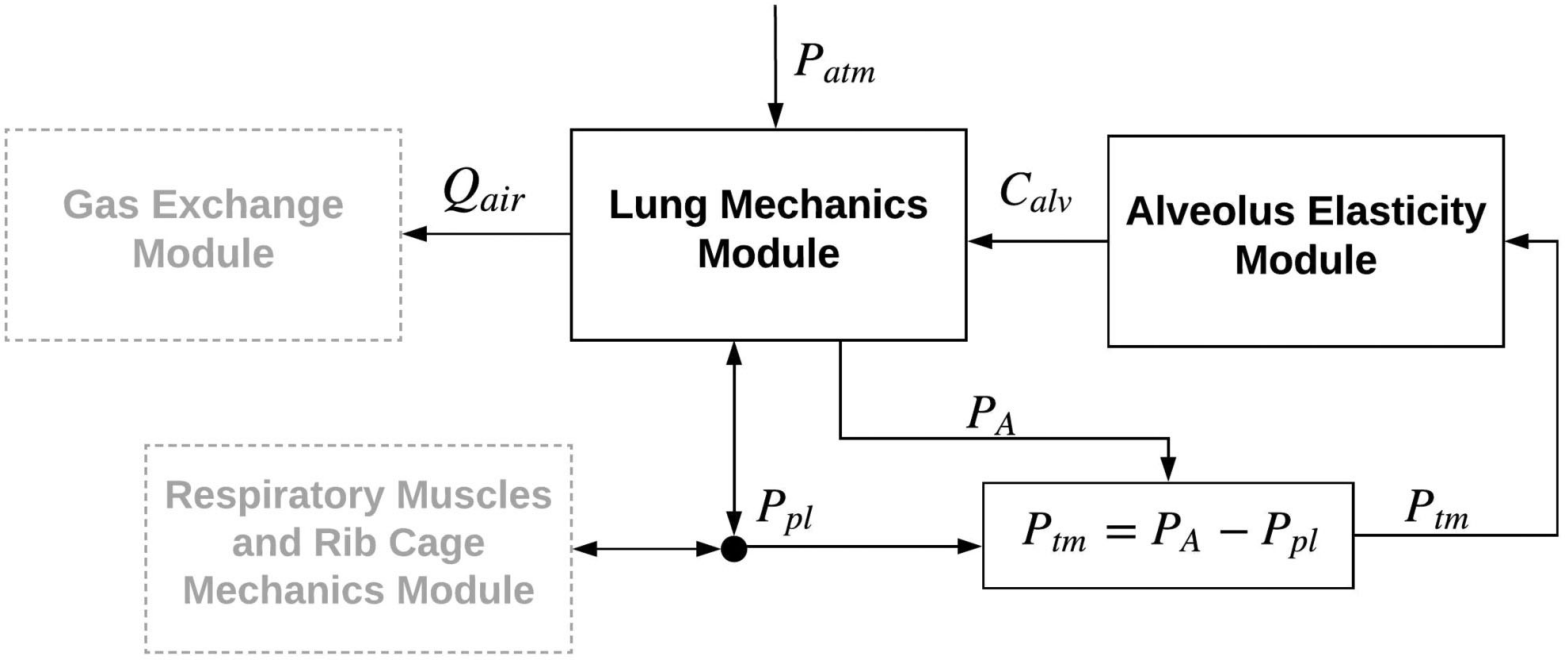
Block diagram of lung modules. The two modules highlighted in black are described in this paper. The full respiratory model comprises: lung mechanics, alveolus elasticity, respiratory muscles and rib cage mechanics, and gas exchange modules. ${P_{atm}}$: atmospheric pressure; ${P_A}$: alveolar space pressure; ${P_{pl}}$: pleural cavity pressure;$\ {P_{tm}}$: transmural pressure; ${C_{alv}}$: alveolar capacitance; ${Q_{air}}$: airflow into and out of the lungs.

A model of the human pulmonary system can be described via four modules, as per Figure 1:

1) A lung mechanics (LM) module that computes airflow (${Q_{air}}$), volumes, and pressures at different lung compartments, such as the alveolar space, as a result of a given pleural cavity pressure (${P_{pl}}$) and an alveolar capacitance (${C_{alv}}$), where ${P_{pl}}$ and ${C_{alv}}$ change in time.
2) An alveolar elasticity (AE) module that quantifies alveolar capacitance as a function of the nonlinear tissue fiber elasticity and the surfactant concentration, both of which change depending on the health of the pulmonary system.
3) A gas exchange module that computes the oxygen and carbon dioxide transport between blood in the pulmonary capillaries and gas in the lungs based on the airflow computed in the LM module.
4) A respiratory muscles and ribcage mechanics module that describes how respiratory muscle contraction affects ribcage motion and pleural cavity pressure.

**Fig. 2.**
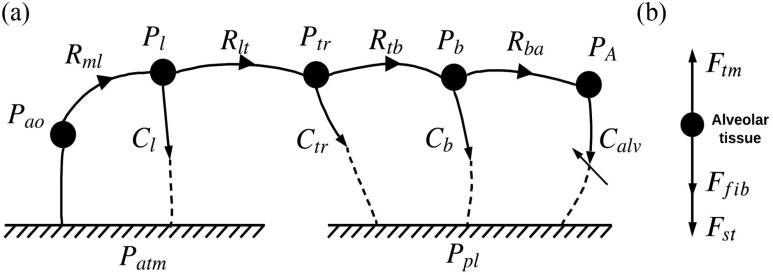
(A) Linear graph of the lung mechanics module, and (B) force balance diagram of alveolar tissue. $ao$: airway opening; $l$: larynx; $tr$: trachea; $b$: bronchi; $A$: alveoli; $pl$: pleural cavity; $atm$: atmosphere; $ml$: mouth to larynx; $lt$: larynx to trachea; $tb$: trachea to bronchi; $ba$: bronchi to alveoli; $tm$: transmural; $st$: surface tension; $fib$: fibers; $P$: hydraulic pressure; F: force; $C$: hydraulic capacitance.

In this paper, we are presenting the first and the second modules only (dark boxes in Figure 1). Following Rideout's work [21], we define four spatial compartments in series, which are larynx, trachea, bronchi and alveoli. A nonlinear module that computes time-varying alveolar capacitance was developed to replace the constant capacitance (or compliance as explained below), used by Rideout. We computed time-varying alveolar capacitance as a function of tissue fiber elasticity and surfactant concentration. Typically, parameters (representing material property and geometry) are constant values and variables, the solutions of the ordinary differential equations, change in time. However, in this work, we computed the time-varying property of the alveolar capacitance. Hence, it is a time-varying parameter. Figure 2A shows the linear graph of the lung mechanics module. We employ this graphing technique to allow for a systematic formulation of the system's dynamic equations [31]. These equations consist of variables and parameters. Parameters represent material property and geometry of the lung compartments, such as hydraulic resistances and capacitors. Pressures and volumes are termed variables, which could potentially be measured through an instrument. In Figure 2A, every node (solid circle) represents pressure within a compartment of the respiratory system. Every line with an arrow represents a flow between two compartments and is labeled with the associated parameter of that segment. Alveolar capacitance (${C_{alv}}$), is indicated with an additional oblique arrow because it is a time-varying parameter that is derived from the AE module. Airway opening (${P_{ao}}$) and larynx (${P_l}$) pressures are referenced to atmospheric pressure. Tracheal (${P_{tr}}$), bronchial (${P_b}$), and alveolar (${P_{alv}}$) pressures are referenced to pleural cavity pressure (${P_{pl}}$) since the pleural cavity anatomically encloses these three compartments. In this model, collagen volume (${V_{col}}$) and surfactant concentration (${\rm{\Gamma }}$) appear as parameters in the AE module equations, since they are variables’ fixed initial conditions that determine the severity of a lung disease in one simulation study, and as such they could be considered like parameters.

The pleural cavity pressure decreases as respiratory muscles contract, as is the case of inspiration, and increases as the pulmonary muscles relax, as in expiration. The reduction in ${P_{pl}}$ generates a positive transmural pressure forcing the alveoli to expand. Alveolar expansion causes an alveolar pressure to drop and creates a pressure gradient between the mouth and the alveoli. Air subsequently gets inhaled into the lungs, and hence we breathe. ${P_{pl}}$ is modeled according to (1) [13]:

\begin{equation*}
{P_{pl}} = \left\{ {\begin{array}{lll} {\ - \ \frac{{{P_{mag}}}}{{{T_I}{T_E}}}{t^2} + \frac{{T \cdot {P_{mag}}}}{{{T_I}{T_E}}}t + {P_{init}}\ \ \ \ \ \ \ \ \ \ 0 \leq t < {T_I}}\\ {\frac{{{P_{mag}}}}{{\left({1 - {e^{ - \frac{{{T_E}}}{\tau }}}} \right)}}\left({{e^{ - \frac{{t - {T_I}}}{\tau }}} - {e^{ - \frac{{{T_E}}}{\tau }}}} \right) + {P_{init}}\ \ \ {T_I} \leq t < T} \end{array}} \right.\tag{1}
\end{equation*}

where ${T_I}$ is the inspiration time, ${T_E}$ is the expiration time, $T$ is the total time for one breath, and $\tau $ is the time constant of the exponential expiratory profile. ${P_{mag}}$ is the magnitude of ${P_{pl}}$ and ${P_{init}}$ is the initial ${P_{pl}}$ value at the beginning of inspiration. During quiet breathing, typically, ${P_{init}}$ is -5 ${\rm{cm}}{{\rm{H}}_2}{\rm{O}}$, ${P_{mag}}$ is 3.5 ${\rm{cm}}{{\rm{H}}_2}{\rm{O}}$, $\tau $ is 0.44 s, when the respiratory rate is 12 breaths/min (bpm), and the I:E ratio (ratio of the inspiratory time to the expiratory time) to 0.6 [13].

From the linear graph in Figure 2A, we can write the dynamic equations to solve for the variables in time at each node by applying continuity and compatibility laws. Continuity equations are derived from the laws of conservation of mass. As an example, 2 represents the larynx pressure node:

\begin{equation*}
{C_l}{\dot{P}_l} = \frac{{{P_{ao}} - {P_l}}}{{{R_{ml}}}} - \frac{{{P_l} - {P_{tr}}}}{{{R_{lt}}}}\tag{2}
\end{equation*}

All the variables in the system of equations change in time but the expression of variables as a function of $t$ has been omitted for clarity. As such ${P_l}$ should really be ${P_l}(t)$, etc. Table I summarizes capacitances ($C$), resistances ($R$), and unstressed volume (${V_u}$) values in the LM module [13] [21], with subscripts, $l$: larynx; $t$: trachea; $b$: bronchi; $alv$: alveoli; $ml$: mouth to larynx; $lt$: larynx to trachea; $tb$: trachea to bronchi; $ba$: bronchi to alveoli.

**TABLE I:** Parameters for Lung Mechanics and Alveolus Elasticity

Lung mechanics parameters			
**Capacitance (L/cmH_2_O)**		Resistance (cmH_2_O $ \cdot {\rm{s}} \cdot {{\rm{L}}^{ - 1}}$)	Unstressed Volume (L)
${{\boldsymbol{C}}_{\boldsymbol{l}}} = 0.00127$ [21]		${R_{ml}} = 1.021$ [21]	${V_{u,l}} = 34.4$ [21]
${{\boldsymbol{C}}_{\boldsymbol{t}}} = 0.00238$ [21]		${R_{lt}} = 0.3369$ [21]	${V_{u,t}} = 6.63\ $ [21]
${{\boldsymbol{C}}_{\boldsymbol{b}}} = 0.0131$ [21]		${R_{tb}} = 0.3063$ [21]	${V_{u,b}} = 18.7$ [21]
${{\boldsymbol{C}}_{{\boldsymbol{alv}}\ }}$**(** (6)**)**		${R_{ba}} = 0.0817$ [21]	${V_{u,alv}} = 1.26$ [13]
**Alveolus elasticity parameters**			
	Septal border (cm^3^)	Cross-linking (hex) (cm^3^)	Cross-linking (square) (cm^3^)
**Elastin**	4.4610×10^-11^	8.6574×10^-11^	9.9960×10^-11^
**Collagen**	6.9616×10^-11^	1.2986×10^-10^	1.4994×10^-10^

The mechanical properties of the alveolar tissue, which determine alveolar capacitance, are a function of fiber (elastin and collagen) stresses and surface tension [32]. At every simulation time step, the alveolus dimension is computed from the quasi-steady state of the force balance equation, ${F_{tm}} - {F_{st}} - {F_{fib}} = 0$, where ${F_{st}}$ is the force due to surface tension, ${F_{fib}}$ is the force due to lung fiber elasticity, and ${F_{tm}}$ is the force due to transmural pressure (${P_A} - {P_{pl}}$), as shown in Figure 2B. Note that this model simulates patients in supine position, where gravity gradient is negligible. The alveolus volume can then be determined by the transmural pressure (${P_{tm}}$) that changes in time. The following describes the empirical relations of the fiber forces that relate fiber force/stress to alveolus dimension, and the assumptions of fiber distribution on a single alveolus.

**Fig. 3.**
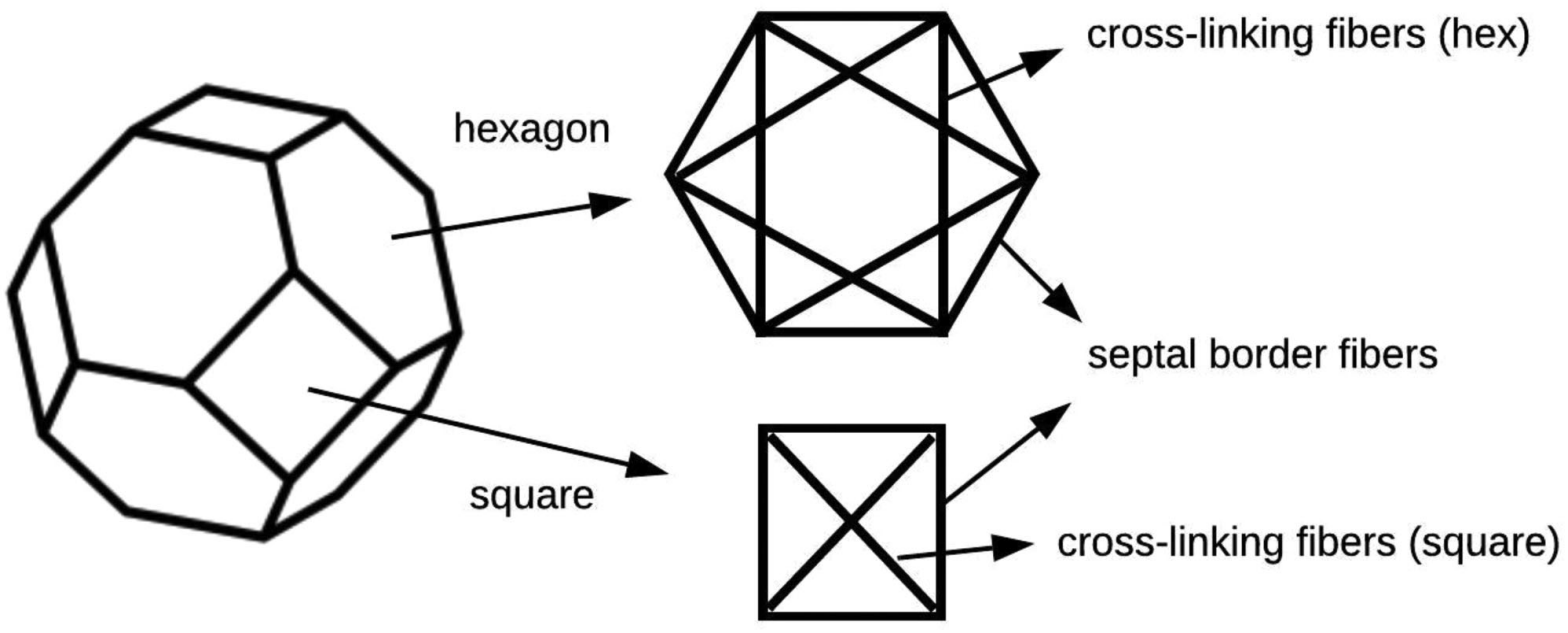
Geometry and fiber structure of a single alveolus.

The elastin fiber is assumed to have a linear stress-strain relation with a Young's modulus of $7.1\ \times {10^6}$ dynes/cm^2^ [25]. The collagen fiber has a highly nonlinear stress-strain relation, as shown in (3):

\begin{equation*}
{\sigma _C} = \ {c_1}\log \left[ {1 - \frac{{\exp ({\epsilon _f}) - 1}}{{{c_2}}}} \right] + {c_3}{\epsilon _f}\tag{3}
\end{equation*}

where ${c_1} = \ - 2.25 \times {10^6}$ dynes/cm^2^, ${c_2} = 1.264$, ${c_3} = \ - 1.78 \times {10^6}$ dynes/cm^2^, and ${\epsilon _f}$ is the fiber strain [25]. In (3), the coefficients of collagen elasticity (${c_1}$, ${c_2}$ and ${c_3}$) can quantify collagen degradation, where ${c_1}$ and ${c_3}$ are coefficients in the first nonlinear and second linear term, respectively, while ${c_2}$ limits strain nonlinearly as a disturbance to the exponential and the log function. In order to compute the fiber force due to fiber elasticity, the volume distribution of elastin and collagen on an alveolus was determined from the following four assumptions: 1) a truncated octahedron is adopted as the shape of one alveolus in the AE module, since Fung *et al.* found that the most common shapes of the surfaces of alveoli were hexagons and rectangles [33]. As shown in Figure 3, we defined septal border fibers and cross-linking fibers on square and hexagonal surfaces following the works by Fujioka *et al.* and by Denny *et al.* [25], [30]. 2) Assuming that the amount of the cross-linking fibers per unit area on a hexagonal face is identical to that on a square face, the volume of the cross-linking fibers on a square face is computed as $\sqrt 2 $ times the volume of same fibers on a hexagonal surface. 3) The alveolus expands and contracts analogously as the inner pressure changes, then the ratio of cross-sectional area between a septal border and a cross-linking fiber bundle is computed as 1.077 [30]. 4) The ratio of the amount of collagen to elastin is 1:5 [30], [34]. The elastin and collagen volume of cross-linking fibers and septal borders are shown in Table I. The fiber forces can thus be computed from the fiber stresses and the cross-sectional area of the fibers.

Surface tension is a function of surfactant concentration as shown in (4):

\begin{equation*}
\gamma = \left\{ {\begin{array}{lll} {{\gamma _0} - E\Gamma \ \ \ \ \ \ \ \ \ \ \ \ \ \ \ \ \ \ \ \ \ \ \ \ \ \ \ \ \ \ \ \ \ \ \ \Gamma < {{\rm{\Gamma }}_\infty }}\\ {{{\rm{\Gamma }}_\infty }\exp \left[ {\frac{E}{{{\Gamma _\infty }}}\left({{\Gamma _\infty } - \Gamma } \right)} \right]\ \ \ \ \ \ \ \ \ \ \Gamma \geq {{\rm{\Gamma }}_\infty }} \end{array}} \right.\tag{4}
\end{equation*}

where ${\rm{\Gamma }}\ $ is the surfactant concentration, Γ_∞_ is a critical surfactant concentration = 3.1×10^-7^ g/cm^2^ [35], ${\gamma _o}$ is basal surface tension = 72 dynes/cm and EΓ_∞_/${\gamma _o}$ = 0.7. ${\rm{\Gamma }}$ is calculated as the ratio of the mass of surfactant to the surface area of an alveolus (i.e., ${m_{surf}}$/${A_{alv}}$). The surfactant mass in one single alveolus is 3.35 × 10^-10^ g [30]. The pressure due to surface tension is determined by Laplace's law.

As ${P_{tm}}$ changes at every time step, the edge length of an alveolus, ${l_{alv}},$ is computed at quasi-steady state using the force balance equation. The volume of an alveolus (truncated octahedron) is computed as $8\sqrt 2 l_{alv}^3$, and surface area of an alveolus is solved as ($6 + 12\sqrt 3)l_{alv}^2$. The alveolar space volume (${V_A}$) is then computed as the product of the number of alveoli and the volume of a single alveolus (${V_{alv}}$). The number of alveoli is set to be 600 million [36]. As such, we have the needed ${V_A}$ that will be used in determining alveolar capacitance.

The fluid (hydraulic) capacitance (${C_f}$) represents a potential energy storage element. It is a combination of three components: open reservoir effects (${C_{reserv}}$), compliance or elasticity effects (${C_{compl}}$), and fluid compressibility effects (${C_{compr}}$), as shown in Equation (5).

\begin{equation*}
{C_f} = {C_{reserv}} + {C_{compl}} + {C_{compr}}\tag{5}
\end{equation*}

During normal breathing, the potential energy storage due to the air compressibility effect is negligible (that is, ${C_{compr}} = 0$), since air pressure in the lungs is low (about one cmH_2_O [37]). Approximating air to an ideal gas, the potential energy storage in the alveolar space due to open reservoir effect (${C_{reserv}}$) is then derived from the ideal gas law, ${C_{reserv}} = {V_A}/\rho RT$, where $R$ is the ideal gas constant, $\rho $ is the density, and $T$ is the temperature. The compliance effect is derived as the ratio between the change of alveolar volume and the change of alveolar pressure, ${C_{compl}} = {\rm{\Delta }}{V_A}/{\rm{\Delta }}{P_A}$. We then compute the alveolar fluid capacitance (${C_{alv}}$) as:

\begin{equation*}
{C_{alv}} = \frac{{{V_A}}}{{\rho RT}} + \frac{{{\rm{\Delta }}{V_A}}}{{{\rm{\Delta }}{P_A}}}\tag{6}
\end{equation*}

Simple calculations show that ${C_{reserv}}$ is two orders of magnitude smaller than alveolar compliance ${C_{compl}}$. Therefore, we conclude that the lung compliance effect serves as the main determinant of potential energy storage in the lungs. As such, we now use the terms capacitance and compliance interchangeably throughout the paper.

The LM module has four 1^st^ order dynamic equations and hence four unknown pressures (at the four nodes in Figure 2A). For non-sedated patients, ${P_{pl}}$ serves as the input of the model. The time-varying alveolar compliance, computed as a function of surfactant concentration (4) and fiber (elastin and collagen) elasticity (3 for collagen and the linear stress-strain function for elastin), is fed into the LM module at every simulation step. The LM variables and the pulmonary conditions (i.e., ARDS and IPF) can thus be simulated as functions of changes in surfactant concentration and lung fiber elasticity.

## III. Results

In this section, we first present the model sensitivity analysis to show the effects of model parameters on the lung mechanics variables. Then, we perform model simulation and validate on healthy non-ventilated subjects and ventilated ARDS or IPF patients, along with the corresponding interpretation and analysis. To validate the model in healthy conditions, we compare the proposed model to 1) an accepted lung model with a constant alveolar compliance value, and 2) measured healthy human flow data. To validate the model in diseased conditions, we compare the model to ARDS and IPF human data. Finally, we assess the robustness and stability of this dynamic system.

### A. Model Sensitivity Analysis

To evaluate the effects of model parameters (collagen volume (${V_{col}}$), collagen elasticity coefficients (${c_1}$, ${c_2}$, and ${c_3}$ from (3)), surfactant concentration (${\rm{\Gamma }}$), hydraulic resistances, and hydraulic capacitances) on the LM variables, we performed a sensitivity analysis of the lung mechanics variables to changes in parameters via sigma (${\rm{\Sigma }}$) values, as shown in Table II. Sigma values were computed to quantify the sensitivity, as $\frac{{{\rm{\Delta }}Variable \times Parameter}}{{{\rm{\Delta }}Parameter \times Variable}}$. The sigma value is a measure of the effect of the change in parameters to changes in variables, where parameters represent material property and geometry of the system (first column of Table II), while variables are the system outputs (first row of Table II). A bigger ${\rm{\Sigma }}$ value indicates a higher sensitivity. Each sigma value is computed using a change in a parameter and corresponding changes in variable. For comparison purposes, we have selected a common range of parameter change in Table II (50% decrease to a 100% increase in 10% increments). The range -50% to 100% corresponds to halving and doubling each parameter, thereby covering a reasonable range to study negative and positive changes of the nominal value. A mean sigma was then generated for each parameter (across all variables) and reported in the cells of Table II. As seen, surfactant concentration is the most sensitive parameter across all lung mechanics variables and has an average sensitivity of 1.17 (computed from the 2^nd^ row). The alveolar elasticity parameter, ${c_2}$, is the second highest sensitive parameter, across all variables, and has an average sensitivity of 0.71. Among the three collagen elasticity parameters (${c_1}$, ${c_2}$, and ${c_3}$) in (3), ${c_3}$ has the least impact on the LM variables, when compared to ${c_1}$ and ${c_2}$. The collagen volume (${V_{col}}$) is the fourth sensitive parameter. The compliances of the upper airways (${C_l}$, ${C_{tr}}$, ${C_b}$) were the least sensitive parameters, followed by some of the resistances (${R_{tb}}$ and ${R_{lt}}$) of the upper airways. This observation was expected since parameters affecting the alveolar compartment (and not the upper airways) are the main determinants of respiratory conditions such as ARDS and IPF, as mentioned in the Introduction, [4]–[9].

**TABLE II:** Sensitivity Results

**Σ**	${P_A}$	${V_A}$	$Q$	${V_{lung}}$	${P_b}$	${P_{tr}}$	${P_l}$
${V_{col}}$	0.114	0.313	0.214	0.294	0.113	0.113	0.112
${\rm{\Gamma }}$	0.929	1.492	1.630	1.396	0.921	0.916	0.915
${c_1}$	0.148	0.242	0.394	0.227	0.146	0.147	0.144
${c_2}$	0.496	1.138	0.826	1.066	0.492	0.490	0.488
${c_3}$	0.038	0.108	0.240	0.101	0.047	0.038	0.036
${R_{ml}}$	0.195	0.074	0.148	0.074	0.217	0.332	0.541
${R_{lt}}$	0.071	0.037	0.074	0.034	0.080	0.121	0.111
${R_{tb}}$	0.069	0.029	0.074	0.027	0.076	0.098	0.097
${R_{ba}}$	0.128	0.081	0.069	0.079	0.144	0.225	0.226
${C_l}$	0.019	0.064	0.022	0.060	0.019	0.018	0.019
${C_{tr}}$	0.037	0.047	0.016	0.045	0.037	0.035	0.036
${C_b}$	0.023	0.068	0.025	0.029	0.024	0.024	0.024

Table II quantifies the sensitivity of the lung mechanics variables to parameter change. ${c_1}$, ${c_2}$, ${c_3}$: coefficients in collagen stress-strain function; ${V_{col}}$: collagen volume; ${\rm{\Gamma }}$: surfactant concentration; ${V_{lung}}$: lung volume; $Q$: total airflow;$\ l$: larynx; $tr$: trachea; $b$: bronchi; $A$: alveoli; ml: mouth to larynx;$\ lt$: larynx to trachea; $tb$: trachea to bronchi; $ba$: bronchi to alveoli; $P$: hydraulic pressure; $C$: capacitance.

**Fig. 4.**
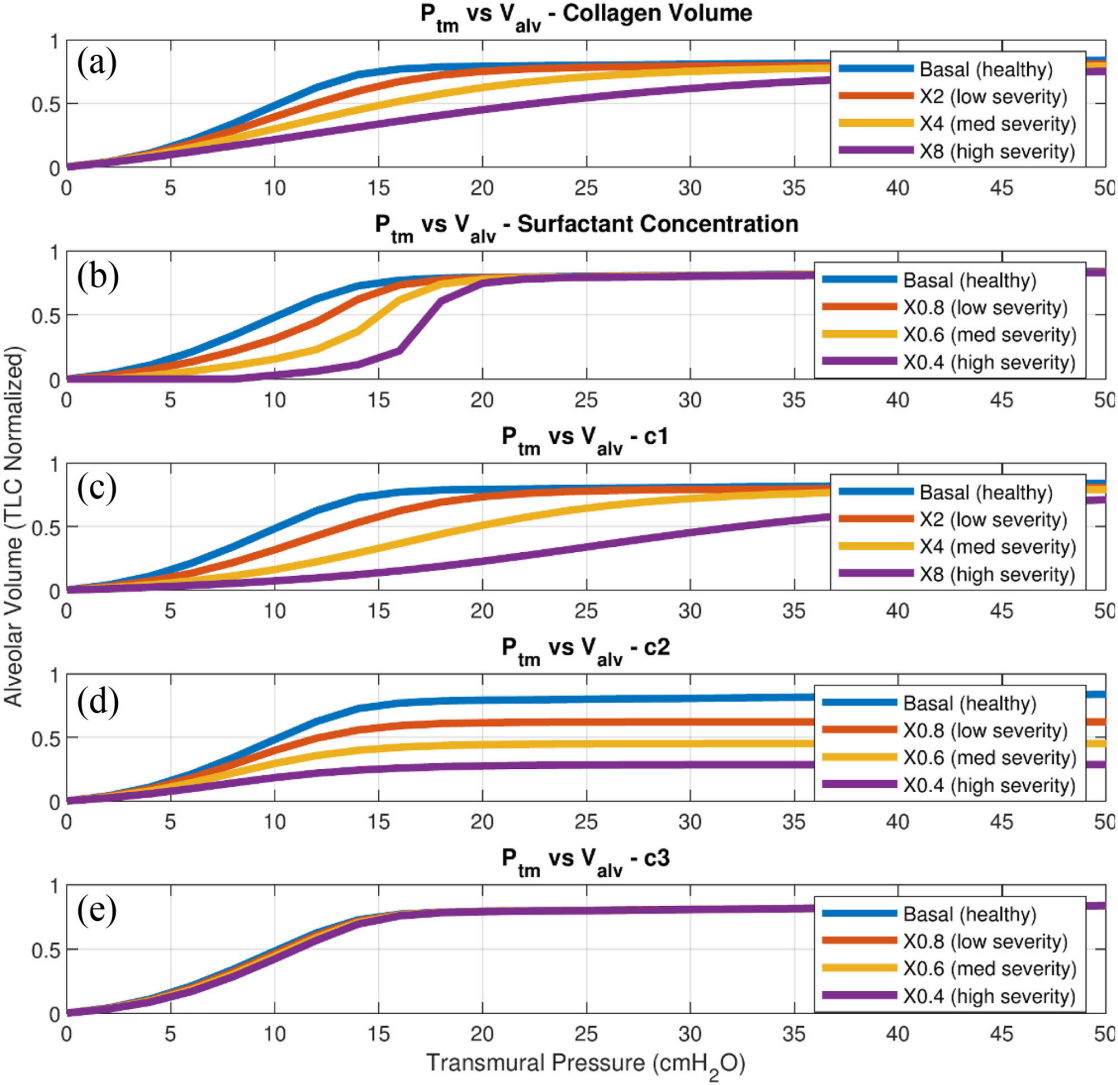
Sensitivity of alveolar volume (total lung capacity (TLC) normalized) and transmural pressure to the change of collagen volume (A), surfactant concentration (B), $c$ values in collagen stress-strain function: 3 (C-E).

Figure 4 shows the effects of the variations of collagen volume, surfactant concentration and $c$ values on static transmural pressure vs alveolar volume (PV) curves in the subplots (Figure 4A-E), respectively. Every curve in Figure 4 represents a severity level that is defined by the magnitude of a parameter change (increase by 2, 4, 6 times, or decrease by 20%, 40%, 60%). The severity levels indicated follow ARDS simulation by Fujioka *et al*. [30]. Alveolar volume in Figure 4 is normalized by total lung capacity (TLC) in order to compare patients with different body weights (lung volume). As reflected in Table II and Figure 4 (subplots C-E), lung pressures and volumes are not sensitive to ${c_3}$ compared to the other collagen elasticity coefficients (${c_1}$ and ${c_2}$). As we analyze the subplots of the sensitivity analysis shown in Figure 4, the subplots A and B reveal that an increase in collagen quantity and/or a decrease in surfactant concentration create stiffer lungs, a fact which agrees with clinical findings [2], [3], [7]–[9]. In Figures 4A and 4C, we find that the inflection points shift to the right as severity level increases. Figure 4B shows that a decrease in surfactant concentration flattens the PV curves, especially in the low-pressure range. Further, the slopes of the curves (compliances) rise faster as surfactant concentration (${\rm{\Gamma }}$) decreases (severity level increases), and all curves reach the same alveolar volume at high pressures. Figure 4B also shows that once pressure exceeds the alveolar threshold opening pressure, the alveoli are open and lung volume increases according to their elastic properties. This threshold opening pressure may be higher for the lower surfactant concentrations, as shown. In Figure 4D, PV curves show high sensitivity of pressures and volumes to changes in ${c_2}$. When ${c_2}$ decreases, not only do the lungs get stiffer (lower slope), but also the maximum alveolar volume is reduced at high ${P_{tm}}$. More interpretation of the sensitivity results can be found in the Discussion section.

Hence from the sensitivity results, LM variables are sensitive to 1) the surfactant concentration (${\rm{\Gamma }}$), 2) the collagen elasticity parameters (${c_1}$, ${c_2}$) in 3, and 3) the collagen volume (${V_{col}}$). Clinical studies also support the fact that ARDS and IPF patients have abnormal ${\rm{\Gamma }}, c$ values and ${V_{col}}\ $ [4]–[9], we thus simulate ARDS and IPF by varying these parameter values.

### B. Simulation and Validation

#### 1) Healthy Subjects

**Fig. 5.**
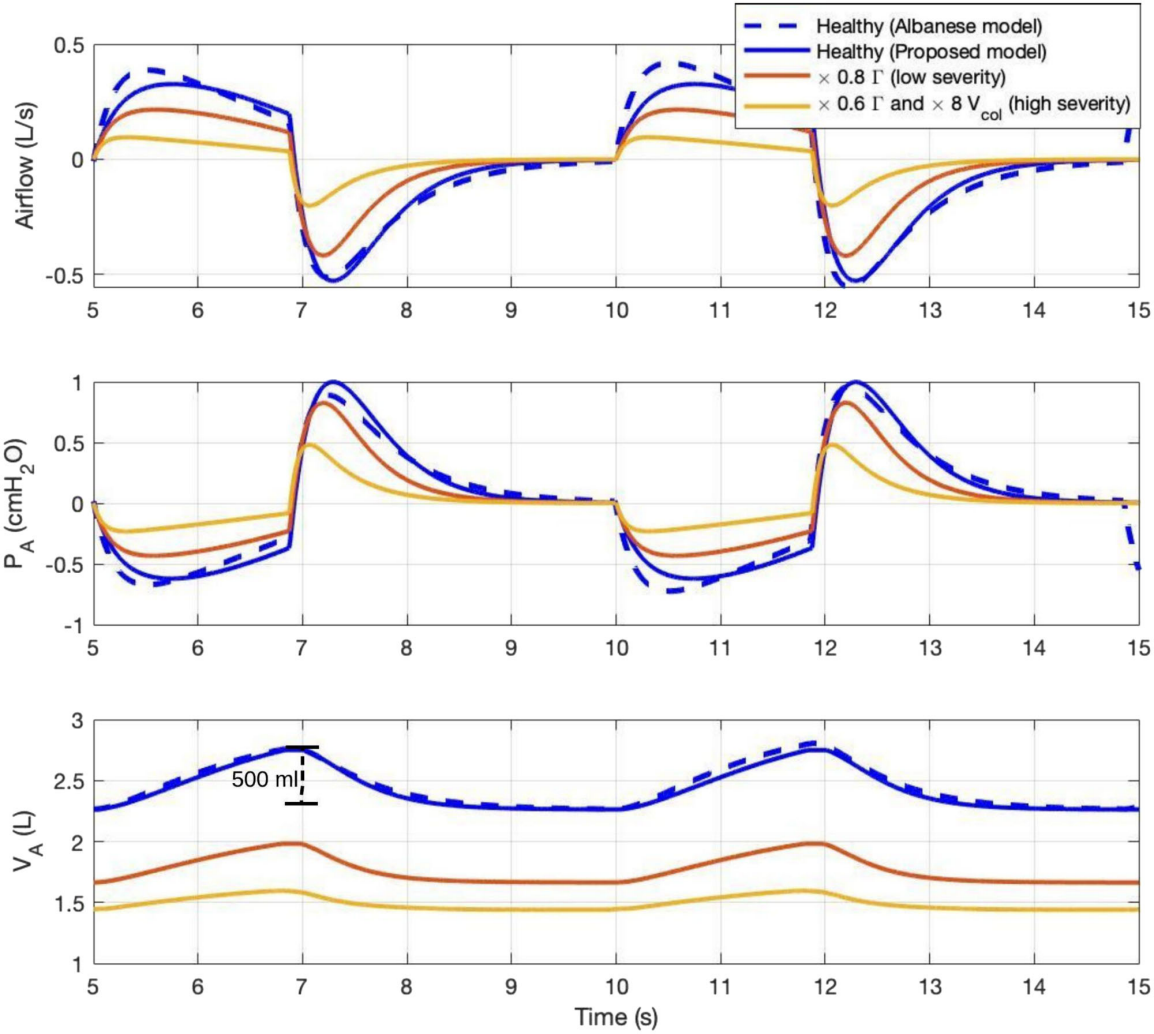
Model simulation of a healthy subject during normal breathing (blue) compared to Albanese's model simulation (blue dashed) [13], and ARDS model simulation with two severity levels (red and yellow): 1) 20% reduction in surfactant concentration, and 2) 40% reduction in surfactant concentration and 8 times more of collagen volume. Airflow, alveolar pressure, and alveolar volume waveforms are shown.

As a first validation step, we compare the proposed model to a published model [13] for a healthy non-ventilated human. Simulation of normal (healthy) breathing is shown in Figure 5(blue curves). The model's lung mechanics variables (solid blue), airflow, alveolar pressure and alveolar volume, are plotted with respect to time and compared to an accepted model (dashed blue) [13]. Our simulation results show that the alveolar pressure becomes negative during inspiration and returns to positive during expiration (varying between -0.6 and 0.98 cmH_2_O). This trend is expected since the airflow follows the pressure gradient between the ${P_{ao}}$ and ${P_A}$ nodes of Figure 2A, as airflow is positive during inspiration and negative during expiration. The lower panel in Figure 5 shows a tidal volume of 500 ml, which agrees with values reported for normal subjects in literature [37]. Our model also reveals a close match to the accepted pulmonary mathematical model (from Albanese *et al*.) [13] — such a model was validated with experimental data from healthy subjects under different environmental conditions [38]. The slight difference between the blue solid and blue dashed curves in Figure 5 is expected since the proposed model adopts a time-varying alveolar compliance, while [13] assumes a constant alveolar compliance of 0.2 L/cmH_2_O. The comparison between the two model simulations in Figure 5 indicates that our proposed model generates waveforms that resemble those predicted by [13]. This observation serves as a preliminary validation of our model.

**Fig. 6.**
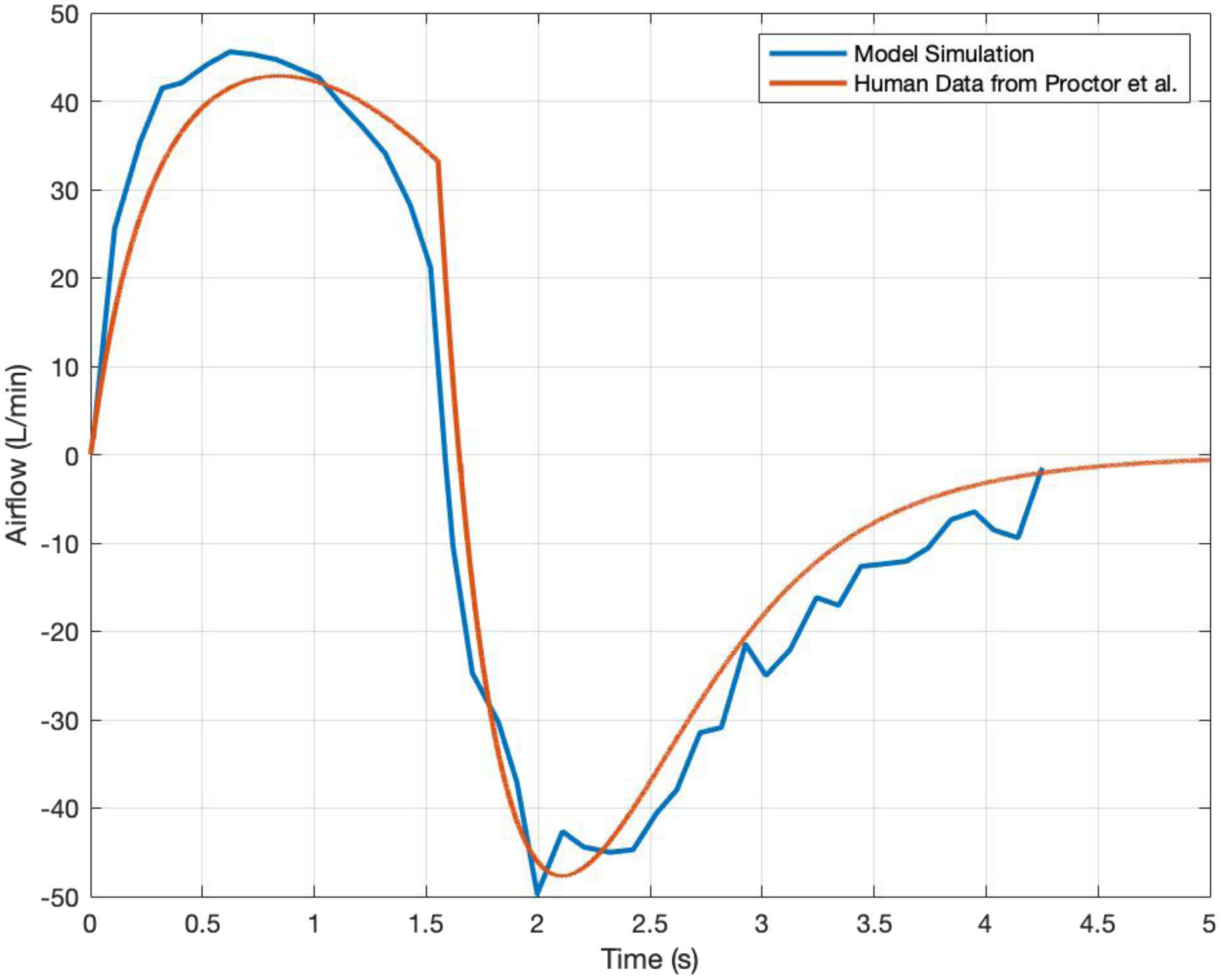
Airflow validated against healthy human data from Proctor *et al.* [12]. Red curve represents healthy human data, and blue curve represent simulation results.

Figure 6 compares our model-simulated airflow to a healthy (non-ventilated) person's airflow as reported by Proctor [12]. In order to match the experimental breathing pattern in [12], we tuned the parameters in (1) to determine the ${P_{pl}}$ profile (the model's forcing function) in order to match the patient flow waveform: we approximated I:E ratio as 0.45, $\tau $ as 0.627 s, and magnitude of ${P_{pl}}$ as 6.5 cmH_2_O. Using this new input and nominal (healthy) parameter values of Table II, our model calculates an airflow waveform that is close to the real human data (root mean squared error: 6.79 L/min). The proposed model emulates healthy patient well since, besides the model input, neither the model parameters nor the equations were changed to fit the human data.

#### 2) Patients With ARDS or IPF

As described in the sensitivity analysis, pulmonary conditions such as ARDS and IPF are greatly affected by, and can be understood as, changes of these four parameters (${\rm{\Gamma }},{V_{col}},\ {c_1},\ {c_2}$) that represent the surfactant concentration, collagen quantity, and collagen quality. In the following sections, we present the simulation results of the time-varying compliance waveforms and the corresponding LM variables under diseased conditions for non-ventilated patients. We then validate our model with ARDS and IPF patient data by tuning these four parameters so the simulation matches experimental results.

**Fig. 7.**
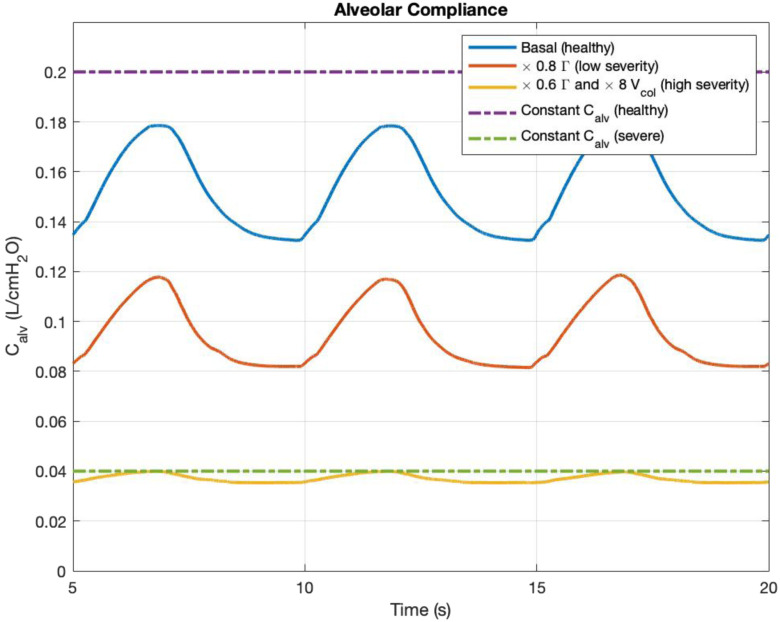
Time-varying alveolar compliance waveforms under two severity levels: 1) 20% reduction in surfactant concentration, and 2) 40% reduction in surfactant concentration and 8 times increase in basal collagen volume. Purple dashed line is the constant alveolar compliance value adopted by Rideout [21]. Green dashed line represents the severe alveolar compliance value in ARDS [39], [40].

In Figure 7, the simulated time-varying compliance waveforms for healthy subjects and diseased nonventilated patients are shown. The solid blue curve represents the simulation of normal subjects whose compliance values oscillate around 0.16 L/cmH_2_O with a magnitude of 0.045 L/cmH_2_O. The purple dashed line represents the constant alveolar compliance that Rideout and Albanese *et al*. adopted in their models [13], [21]. They reported similar LM variable waveforms as ours, as presented in Figure 4. The red and yellow solid curves are the compliance waveforms simulation, using the parameter change for ARDS patients from Fujioka *et al*. [30]. The red curve (low ARDS severity) has 20% reduction in surfactant concentration, and the yellow curve (high ARDS severity) has 40% reduction in surfactant concentration as well as 8 times increase in basal collagen volume. The ARDS simulation with a high severity level generates a compliance curve that barely oscillates since the lungs are much less elastic. The yellow compliance waveform reaches a value close to 0.04 L/cmH_2_O, which matches the severe compliance reported in the literature as shown in the green dashed curve [39], [40]. Note that the simulated time-varying compliance shown in Figure 7 is bounded between the static healthy and diseased compliance values from literature, further supporting the simulation results.

The resultant LM variables in time (airflow, alveolar pressure, and alveolar volume) with time-varying compliances are shown in Figure 5 for healthy subjects (blue) and diseased nonventilated subjects (red and yellow curves). As alveolar compliance decreases (disease severity level increases), all LM variables exhibit peak-to-peak decreases. Tidal volumes are reduced and airflow and pressure reach lower peaks as compared to healthy patient simulation. The tidal volume reduced to approximately 150 ml from a normal value of 500 ml (77% reduction). This is expected due to the increased stiffness of the lungs. The low tidal volume in the high-severity case also indicates the need for exogenous ventilation.

**Fig. 8.**
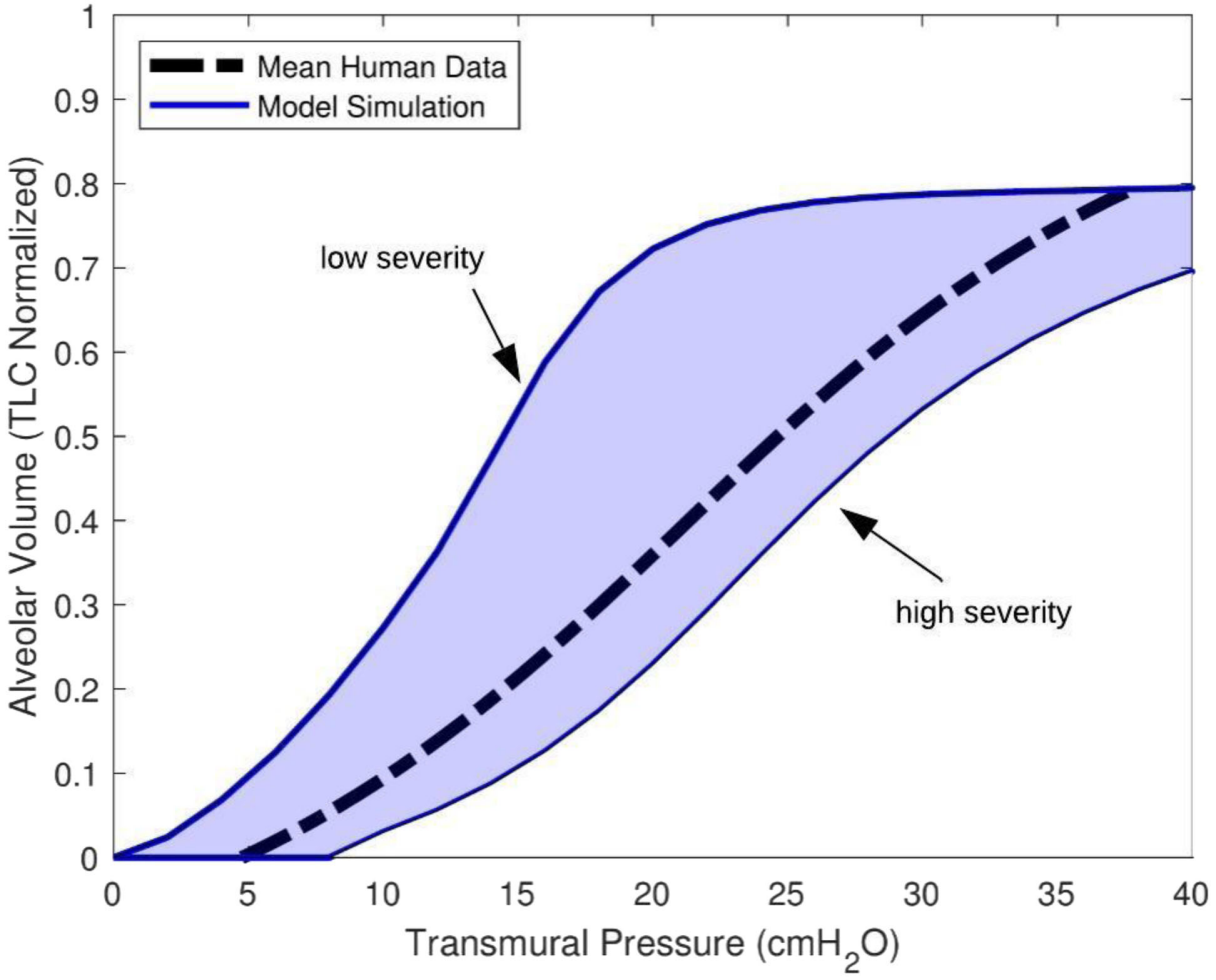
Model validation against mean PV data from 23 ARDS patients (dashed curve). Two border lines were simulated with low and high severity levels as defined in sensitivity analysis. The blue shadow covers the area of general ARDS PV curve data according to our simulation.

ARDS human data from three different studies [14]–[16] were obtained to validate our model. All patients were fully sedated and intubated with mechanical ventilator support. Orfao *et al*. [15] reported a mean PV curve from 23 ARDS patients, plotted as the dashed black line in Figure 8. The reported lung volume is normalized by total lung capacity (TLC), which was estimated from the sigmoidal fitting function: $V = \ a + \frac{b}{{1 + {e^{ - ({P\; -\; c})/d\ }}\ \ }}$, where $a,\ b,\ c,\ {\rm{and}}\ d$ are four fitting parameters. The TLC can be estimated from $a + b$ or read from the upper asymptote by considering the pressure interval from 0 to 100 [15]. The sigmoidal function has been shown to fit the PV curve, and it is a well-accepted approximation for understanding the lung mechanics of ARDS patients when appropriately tuned [15], [23]. The transmural pressure in Figure 8 is determined by the difference between ${P_A}$ and ${P_{pl}}$. The pleural cavity pressure shows positive swings as the ventilator blows air into the lungs tidally. Assuming nominal chest wall compliance (${C_{cw}}$) of 0.2445 L/cmH_2_O [13], pleural cavity pressure is equal to $\frac{{{V_A}}}{{{C_{cw}}}}$. Using the low and the high severity defined in Figure 5, we generated two independent PV curves (blue solid) that envelop the mean PV curve. This is expected since the two border PV curves are determined with the extreme parameter change following the work by Fujioka *et al.* [30].

**Fig. 9.**
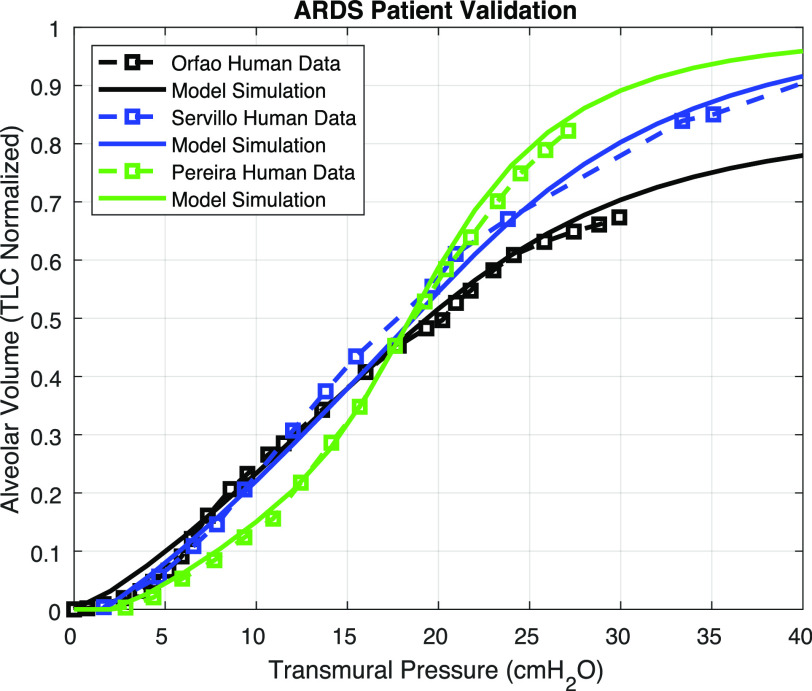
Model validation against ARDS patient data from three references [14]–[16]. Dashed lines represent ARDS patient data, and solid lines represent model simulation results.

Orfao, Servillo, and Pereira [14]–[16] each reported PV data for one ARDS patient, plotted in black, blue, and green dashed lines in Figure 9, respectively. The sensitive and disease-important parameters for the ARDS patients, namely ${\rm{\Gamma }}$ and ${V_{col}}$, are determined via exhaustive search to match the literature-reported PV curves. Once the parameters are estimated, we fix the set of the disease-related parameters, and generate this patient's PV curve from the model to compare to the data reported in literature. The ${R^2}$ values computed from comparing the model-simulated and literature-reported PV curves are reported in Table III along with the corresponding parameter scaling factors applied to fit the model to the data. The model approximates the physical data reasonably well. In Figure 9, the simulated PV curves with the estimated parameters also agree with the sensitivity analysis (Figure 4). As noted, Pereira's ARDS data is flatter at low pressure (steeper S-shaped) than the other two (Orfao's, Servillo's) PV curves, indicating a reduced surface tension effect, as learned from Figure 4B. As a result, a greater reduction of ${\rm{\Gamma }}$ was indeed needed to emulate the Pereira patient data, as compared to the Orfao or Servillo patient data. The estimated parameter variations of the three ARDS patients are reasonable since the scaling factors are between the low and high severity as defined earlier. Also, the collagen volume change in ARDS patients were quantified by Saldiva *et al*. [41]. In their study, the color intensity of stained lung tissue showed that collagen volume of ARDS patients can increase by 2.7 times (and more than 10 times for a severe case) compared to a normal patient group on average.

**TABLE III:** Parameter Scaling Factors for Ards and Ipf Patients

ARDS Patients							
**Patient References**		${V_{col}}$		${\rm{\Gamma }}$			R^2^
**Orfao** [15]		6		0.9			0.9973
**Servillo** [14]		6.5		0.8			0.9937
**Pereira**[16]		4		0.58			0.9794
IPF Patients							
Patient ID	${c_1}$		${c_2}$		${V_{col}}$	R^2^	
Ferreira [17] - 1	8		1		1	0.9907	
Ferreira [17] - 2	0.7		0.5		6	0.9949	
Ferreira [17] - 3	0.7		0.61		4.5	0.9943	
Ferreira [17] - 4	2.4		0.78		4	0.981	
Ferreira [17] - 5	1.8		0.5		8	0.992	
Ferreira [17] - 6	0.3		0.75		1.6	0.9926	

R^2^: coefficient of determination; ${c_1}$, ${c_2}$: coefficients in collagen stress-strain function; ${V_{col}}$: collagen volume; ${\rm{\Gamma }}$: surfactant concentration.

**Fig. 10.**
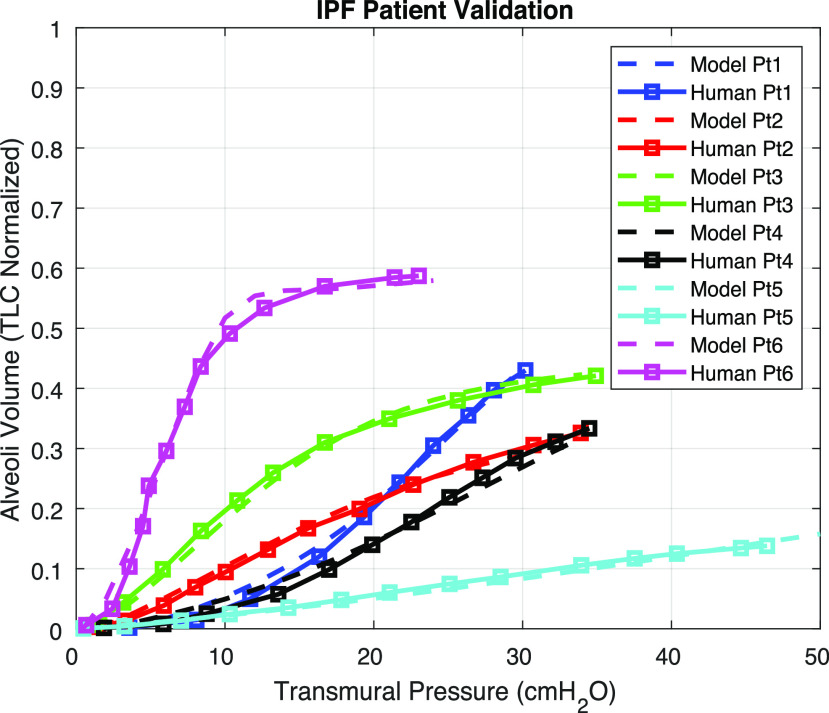
Model validation against six IPF patients [17]. Solid lines represent real IPF patient data, and dashed lines represent model simulation results.

The proposed model is also employed to compute pulmonary elasticity (PV curves) of IPF patients. Six IPF patients’ PV curves were reported in [17]. All patients were fully anesthetized and intubated with mechanical ventilator support. IPF is a disease resulting from collagen degradation and increase in quantity. Since ${c_3}$ does not greatly affect lung mechanics variables, according to the Sensitivity Analysis, and the IPF pathophysiology does not support a decrease in ${\rm{\Gamma }}$ for IPF patients, we simulate IPF by exhaustively searching for the optimal ${c_1}$, ${c_2}$, and collagen volume only. To compare different patients and to compare patient data to model simulation, we normalized the reported lung volumes by TLC values. Table III summarizes the multiplicative factors applied to the healthy parameters and ${R^2}$ values from comparisons of model-simulated and literature-reported PV curves. A multiplicative factor of 1 means that either the nominal or the healthy parameter value was used. Figure 10 shows all six patients’ data along with our simulation results. Our model-simulated PV curves match the six patients’ data well. The model simulates IPF data of patient 1 by an eightfold increase in ${c_1}$. This result agrees with the sensitivity analysis, as the first order derivative of the patient's PV curve is monotonically increasing, which is similar to the effect of altering ${c_1}$ in Figure 4C. This result also agrees with findings by Fulmer [8] that certain IPF patients do not have an increase in collagen volume in the lungs. Other IPF patients require a combination of both the elastic properties of collagen and its volume. For example, the PV curve of patient 2 is flatter and the total lung capacity is low even at high pressures. This implies a greater increase in collagen volume (as seen in Figure 4A) and a reduction in ${c_2}$ (as seen in Figure 4D), which agrees with the estimated parameters. The alterations in collagen volume that emulate the reported patients’ data also fall in the ${V_{col}}$ range as reported by Saldiva *et al*. [41]. Saldiva *et al*. reported that IPF patients have an average of 3.9 times increase in the collagen volume compared to a normal patient group, and a severe case can have an increase of more than 10 times.

### C. Model Stability and Robustness Assessment

**Fig. 11.**
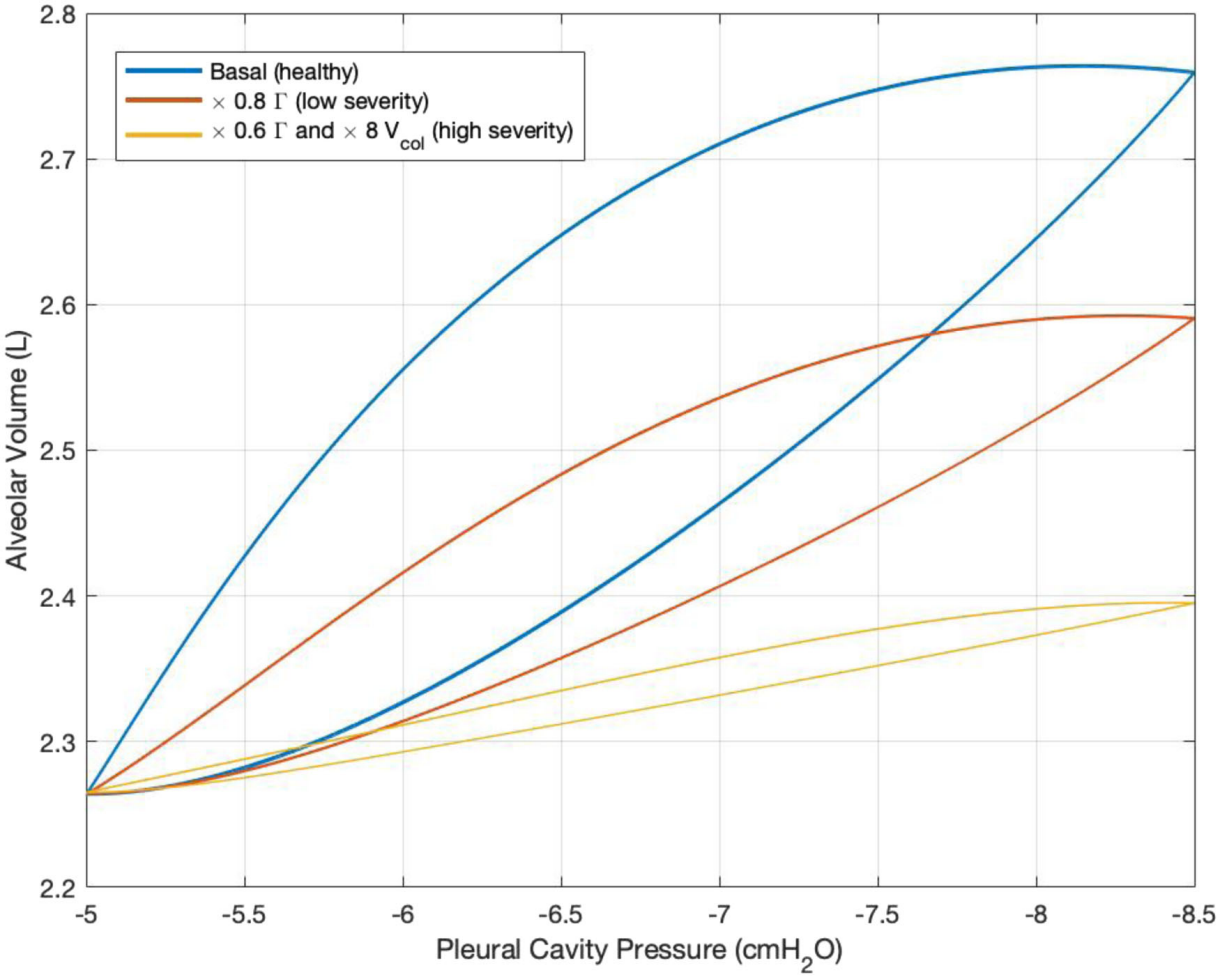
PV loops for long-time breathing simulation in healthy (blue), low severity (red) and high severity (yellow) sickness levels. Low severity: 20% reduction in surfactant concentration. High severity: 40% reduction in surfactant concentration and 8 times increase in basal collagen volume.

To assess the stability and robustness of the proposed model, we perform an eigenvalue analysis, generate phase plane plots for unperturbed and perturbed long-time simulations, and assess feasible parameter ranges. To prove the dynamic stability, we first linearized the time-varying ${C_{alv}}$ in order to formulate the dynamic system into a state-space form, and the state equation can be found in the Appendix. The computed eigenvalues of the state matrix all have negative real parts, namely, $\ - 4631.8$, $ - 1579.4$, $ - 740.2$, and $ - 2.7$, indicating stability of the linearized model. For numerical stability we have simulated the model for more than 4000 breaths (300+ simulation hours) on a 2.9GHz 8GB machine and plotted the PV loops for healthy (blue), low severity (red) and high severity (yellow) levels as shown in Figure 11. The system output loops (pleural cavity pressure vs alveolar volume) are closed, indicating a well-behaved system under both healthy (unperturbed) and diseased (perturbed) cases. Through these analyses, system stability is maintained when multiplicative factors perturbing the parameters (representing lung diseases) are bound by the following limits: ${V_{col}} > 0$, ${\rm{\Gamma }} \geq 0$, ${c_1} > 0$, and $0 < {c_2} \leq $ 1. Note that physiological systems typically have positive-only parameters, since negative parameter values do not have physical meaning.

## IV. Discussion

The lung is often modeled as an *RC* circuit, where *R* represents the hydraulic resistance and *C* represents the compliance of the whole respiratory system. Such a model can be used to describe dynamically the lung pressure and flow reasonably well, by first assigning values for *R* and *C*, and then solving, a system of 1^st^ order ordinary differential equations (ODEs), for the pressure and flow in time. Disease simulation is then accomplished via changing the parameters *R* and *C* and again solving the ODEs. In such simulations, ARDS, IPF, and other compliance-compromised conditions would all be modeled in a similar fashion, e.g., change *C* and then solve for flow and pressure. In the present work, however, ARDS, IPF, and other compliance-compromised conditions can be modeled through the mechanisms that cause compliance changes, such as collagen remodeling or surfactant degradation. In this way, a deeper level of understanding of respiratory dynamics is achieved through more rigor in the model.

In this study, we presented a mechanistic model of the respiratory physiology, specifically how alveolar tissue fibers and surfactants affect lung compliance and breathing. The model validated reasonably well against ARDS and IPF patient data demonstrating its possible use to run what-if scenarios simulating lung conditions and diseases. Interestingly, through simulations of severe disease, we find that lung volumes are extremely low, indicating the need for interventional ventilatory support. Additionally, PV curves of severe disease simulations (increased collagen, decreased elasticity, decreased surfactant) have inflection points that are shifted to the right, indicating stiffer lungs and a greater pressure required to achieve the same volume. In some cases, such as severely reduced surfactant concentration, the PV curve remains nearly flat at low pressure levels, suggesting that more pressure is required to overcome the alveoli threshold opening pressure and supporting the use of high PEEP (positive end-expiratory pressure) to prevent alveolar collapse in ventilating ARDS patients [42].

Through the sensitivity analysis presented, we have confirmed that parameters that determine the health of the alveolar space, such as surfactant concentration (${\rm{\Gamma }}$) and collagen fiber properties ($c$ values and ${V_{col}}$), have a greater impact on lung mechanics variables (lung pressure, flow, and volume) than resistances and compliances of the upper airways. The role of these important parameters is supported in the literature. In ARDS, excess fluid accumulation in the lungs affects the concentration of pulmonary surfactant significantly, which causes alveolar collapse, especially at low pressure ranges [4]. ARDS has also been shown to cause an increase in collagen volume [5], [6]. IPF, on the other hand, which is characterized by scarring and destruction of the lung architecture, tends to be a chronic disease with an excessive increase of collagen volume [43], and polymorphism [7]–[9]. Our model can differentiate between ARDS (via ${\rm{\Gamma }}$ and ${V_{col}}$) and IPF (via ${c_1}$, ${c_2}$, and ${V_{col}}$) since all these parameters appear explicitly therein.

Further, with the linearized version of this model and system identification techniques, we can estimate not just compliance changes but fiber or surfactant properties that caused these changes. In this way, the model can also simulate some COVID patients who resemble ARDS patients in that they have compromised compliance. According to Gattinoni [11], 20-30% of the COVID patients admitted to the intensive care unit have severe hypoxemia associated with low compliance values. These COVID patients with compromised compliance can potentially be simulated via this model. However, this model may not generalize well to patients who have near-normal pulmonary compliance with isolated viral pneumonia [11]. Further studies are warranted.

While we present a time-varying compliance in this work, we have not yet modeled the development of ARDS or IPF in time, which may be of importance in ARDS, as the lungs often show signs of fibrosis or fibrotic scarring in late or severe stages [5]–[6]. However, with real-time parameter estimation we may be able to continually estimate these parameters to assess how they are changing and how the condition is progressing or deteriorating. Furthermore, though the model satisfies the need to understand compliance change during a breath cycle, its effect on LM variables is more prominent in diseased lungs than it is in healthier ones.

## V. Conclusion

In this paper, we have developed a mathematical model of the lung mechanics comprising alveolar tissue and surfactant properties that generates reasonable lung pressures and volumes when compared to healthy, ARDS, and IPF patient data. The model describes a time-varying alveolar compliance that provides a better understanding of lung diseases. We have also shown, through sensitivity analysis, that the surfactant concentration and the collagen stiffness parameter ${c_2}$ have a strong impact on lung mechanics variables. Further, the model has proven to be stable and robust under different disturbances.

The model is a set of ODEs that can be implemented to allow for what-if scenario testing via changing specific parameters. Using measurements for patient and a parameter estimation technique a personalized version of the model can be obtained. The research team is working toward model simulations that test different ventilation strategies for a specific patient, e.g., varying ventilator settings (pressure and PEEP) to simulate the change of airflow, lung pressure and volume of that patient.

## Appendix

The state equation of the linearized model is shown below:

\begin{align*}
\begin{array}{l} \left[ {\begin{array}{c} {{{\dot{P}}_l}}\\ {{{\dot{P}}_t}}\\ {{{\dot{P}}_b}}\\ {{{\dot{P}}_A}} \end{array}} \right] = \\
\left[\! {\begin{array}{cccc}
- \frac{1}{{{C_l}{R_{ml}}}} - \frac{1}{{{R_{lt}}{C_l}}} &{\frac{1}{{{R_{lt}}{C_l}}}}&0&0\\
{\frac{1}{{{R_{lt}}{C_t}}}}& - \frac{1}{{{C_t}{R_{lt}}}} - \frac{1}{{{R_{tb}}{C_t}}} &{\frac{1}{{{R_{tb}}{C_t}}}}&0\\
0&{\frac{1}{{{C_b}{R_{tb}}}}}& - \frac{1}{{{C_b}{R_{tb}}}} - \frac{1}{{{R_{ba}}{C_b}}} &{\frac{1}{{{R_{ba}}{C_b}}}}\\
0&0&{\frac{1}{{{R_{ba}}{C_{alv}}}}}&{ - \frac{1}{{{R_{ba}}{C_{alv}}}}} \end{array}} \! \right]\\
\left[ {\begin{array}{l} {{P_l}}\\ {{P_t}}\\ {{P_b}}\\ {{P_A}} \end{array}} \right] + \left[ {\begin{array}{cc} {\frac{1}{{{R_{ml}}{C_l}}}}&0\\ 0&1\\ 0&1\\ 0&1 \end{array}} \right]\left[ {\begin{array}{c} {{P_{atm}}}\\ {{{\dot{P}}_{pl}}} \end{array}} \right] \end{array}
\end{align*}
